# Impact of a synbiotic food on the gut microbial ecology and metabolic profiles

**DOI:** 10.1186/1471-2180-10-4

**Published:** 2010-01-07

**Authors:** Beatrice Vitali, Maurice Ndagijimana, Federica Cruciani, Paola Carnevali, Marco Candela, Maria Elisabetta Guerzoni, Patrizia Brigidi

**Affiliations:** 1 Department of Pharmaceutical Sciences, University of Bologna, Bologna, Italy; 2 Department of Food Science, University of Bologna, Bologna, Italy; 3 R&D Food Microbiology & Bioprocess Research, Barilla G&R f.lli SpA, Parma, Italy

## Abstract

**Background:**

The human gut harbors a diverse community of microorganisms which serve numerous important functions for the host wellbeing. Functional foods are commonly used to modulate the composition of the gut microbiota contributing to the maintenance of the host health or prevention of disease. In the present study, we characterized the impact of one month intake of a synbiotic food, containing fructooligosaccharides and the probiotic strains *Lactobacillus helveticus *Bar13 and *Bifidobacterium longum *Bar33, on the gut microbiota composition and metabolic profiles of 20 healthy subjects.

**Results:**

The synbiotic food did not modify the overall structure of the gut microbiome, as indicated by Polymerase Chain Reaction-Denaturing Gradient Gel Electrophoresis (PCR-DGGE). The ability of the probiotic *L. helveticus *and *B. longum *strains to pass through the gastrointestinal tract was hypothesized on the basis of real-time PCR data. In spite of a stable microbiota, the intake of the synbiotic food resulted in a shift of the fecal metabolic profiles, highlighted by the Gas Chromatography Mass Spectrometry Solid Phase Micro-Extraction (GC-MS/SPME) analysis. The extent of short chain fatty acids (SCFA), ketones, carbon disulfide and methyl acetate was significantly affected by the synbiotic food consumption. Furthermore, the Canonical discriminant Analysis of Principal coordinates (CAP) of GC-MS/SPME profiles allowed a separation of the stool samples recovered before and after the consumption of the functional food.

**Conclusion:**

In this study we investigated the global impact of a dietary intervention on the gut ecology and metabolism in healthy humans. We demonstrated that the intake of a synbiotic food leads to a modulation of the gut metabolic activities with a maintenance of the gut biostructure. In particular, the significant increase of SCFA, ketones, carbon disulfide and methyl acetate following the feeding period suggests potential health promoting effects of the synbiotic food.

## Background

Humans can be considered as "superorganisms" with an internal ecosystem of diverse symbiotic microorganisms and parasites that have interactive metabolic processes. Their homeostatic balance is dependent upon the interactions between the host and its microbial components [1]. The human intestine is home to some 100 trillion microorganisms of at least 1000 species. The density of bacterial cells in the colon has been estimated at 10^11 ^to 10^12 ^per ml, which makes it one of the most densely populated microbial habitats known [2,3]. This microbial ecosystem serves numerous important functions for the human host, including protection against pathogens, nutrient processing, stimulation of angiogenesis, modulation of intestinal immune response and regulation of host fat storage [4,5]. The composition of the adult gastrointestinal microbiota has been intensely studied, using both cultivation and, more recently, culture-independent, small subunit (SSU) ribosomal DNA (rDNA) sequence-based methods [6-8]. Members of the anaerobic genera *Bacteroides*, *Eubacterium*, *Clostridium*, *Ruminococcus*, and *Faecalibacterium *have typically been found to comprise a large majority of the human adult gut microbial community. In healthy adults, the gut microbiota consists of a stable individual core of colonizing microorganisms surrounded by temporal visitors [9,10]. Fluctuations around this core of phylotypes are due to host genotype, diet, age, sex, organic disease and drugs (especially antibiotics) [11]. It has been shown that the microbiota structure strongly influences the gut metabolic phenotype [12,13]. On short time scales, the host-specific effects are relatively constant and changes in the gut microbiome composition and activities are closely influenced by dietary variations.

An increasing awareness of the potential of gut microorganisms to influence human health has led to widespread investigation of the relationship between the gut microbiota and nutrients, particularly probiotics [14] and prebiotics [15] and their impact on the digestive system. Members of the genera *Bifidobacterium *and *Lactobacillus*, natural components of the colonic microbiota, are the most commonly used probiotic bacteria in many functional foods and dietary supplements [16]. Postulated health advantages associated to bifidobacteria and lactobacilli include the inhibition of pathogenic microorganisms, improvement of lactose digestion, reduction of serum cholesterol levels, prevention of cancer and enhancement of the host's immune system [17,18]. Several oligosaccharides have been studied as potential prebiotics, including lactulose, galactooligosaccharides and fructooligosaccharides (FOS) [19]. Dietary supplements of prebiotics increase the content and proportion of bifidobacteria [20] and exert positive effects on absorption of nutrients and minerals, synthesis of vitamins, prevention of constipation, colon cancer, and improvement of blood sugar and lipid profile [21]. Another possibility in the microbiota modulation is the use of synbiotics, in which probiotics and prebiotics are used in combination. This combination improves the survival of the probiotic strains, because specific substrates are readily available for their fermentation, and results in advantages to the host that the live microorganisms and prebiotics offer [11].

The inadequacy of conventional culture techniques to reflect the microbial diversity of the intestinal ecosystem has triggered the development of culture-independent 16S rRNA gene-based techniques for the evaluation of the effects of functional food administration in humans [22,23]. The latest frontier in the characterization of uncultured and complex microbial communities is the high-throughput technology of pyrosequencing, which achieves hundreds of thousands of sequences of a specific variable region within the small subunit of rRNA gene, consequently revealing the full diversity of an ecosystem [24,25]. However, since this approach is extremely labor intensive and time consuming, PCR-DGGE and real-time PCR represent population fingerprinting methods, commonly used to analyze the intestinal microbiota upon dietary intervention. PCR-DGGE allows the visualization of the predominant genetic diversity without prior knowledge of the composition or complexity of the microbial ecosystem present in the sample [23,26]. Real-time PCR enables specific intestinal bacterial populations to be directly quantified by using DNA isolated from fecal material [23,27-29].

Gene expression profiling and proteomic approaches have been applied to elucidate the molecular mechanisms underlying symbiotic host-bacterial relationships [30-32]. However, gene expression and proteomic data might only indicate the potential for physiological changes because many pathway feedback mechanisms are simply not reflected in protein concentration or gene expression. On the other hand, metabolite concentrations and their kinetic variations in tissues or biological matrixes represent real end-points of physiological regulatory processes [1,33]. Metabonomics is defined as "the quantitative measurement of the dynamic multiparametric metabolic response of living systems to pathophysiological stimuli or genetic modification" [34]. Metabonomics provides a systems approach to understand global metabolic regulation of an organism and its commensal and symbiotic partners [1]. Recently, complementary metabonomic approaches have been employed for the biochemical characterization of metabolic changes triggered by gut microbiota, dietary variation and stress interactions [35-39]. Solid phase microextraction followed by gaschromatography and mass spectrometry represents a novel method for studying metabolic profiles of biological samples. This approach has been used to compare neonates and adult feces [40] and to identify volatile markers of gastrointestinal disease [41].

In the present study, we characterized the impact of the intake of a synbiotic snack on the gut microbiota composition and metabolic profiles of healthy subjects. The synbiotic snack contained the substrate FOS, whose prebiotic effects are widely documented [42], and the probiotic strains *Lactobacillus helveticus *Bar13 and *Bifidobacterium longum *Bar33, which were selected on the basis of their adhesion and immune-regolation properties, as assessed by both in *vitro *[43] and *in vivo *studies on animal models [44]. Co-variations were searched between the gut microbiome structure, as reflected by community DNA fingerprints derived from PCR-DGGE and real-time PCR data, and host metabolic phenotypes, as detected by GC-MS/SPME.

## Results

### Effects of the synbiotic food on composition of the gut microbiota

**Figure 1:**
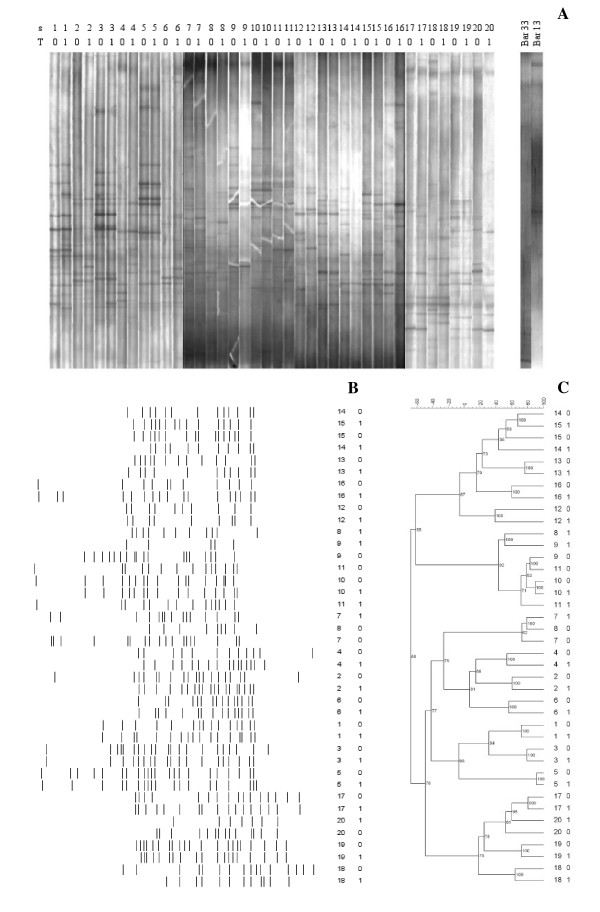
**DGGE analysis of the fecal samples recovered from 20 healthy volunteers (s1-s20) before (T0) and after (T1) one month of the synbiotic intake**. A: DGGE profiles related to fecal samples and *L. helveticus *Bar13 and *B. longum *Bar33 probiotic strains. B: line graph. C: Cluster analysis (Pearson correlation was used to calculate the similarity in DGGE profiles).

PCR-DGGE analysis with universal primers targeting the V2-V3 region of the 16S rRNA gene was used to monitor the impact of the synbiotic food intake on the predominant bacterial population (Figure 1A). Population fingerprint profiles were compared and numerically analyzed by FPQuest Software. DGGE band profiles (mean of bands: 15.3) were stable for each subject over a month of feeding with the functional food. Only a slight difference in band richness was found between the time points of the study (T0, mean of bands: 15.8; T1, mean of bands: 14.8). DGGE bands were subjected to Mann-Whitney U-test in order to search for significant differences in the intensities between T0 and T1. No band showed a significant variation, indicating that the consumption of the synbiotic food did not alter the concentration of any major species of intestinal microbiota. Pearson correlation was used to calculate the similarity index (SI) between DGGE band profiles related to the time points T0 and T1 for each healthy volunteer (Table 1). The high median value of SI (67.1%) revealed that the dominant bacterial composition remained constant over the treatment. Only 3 subjects presented SIs lower than 50% (subjects 8, 12 and 20). No subject showed significant variations between DGGE band profiles related to T0 and T1, as evaluated using the Pearson correlation analysis (*P *> 0.05).

**Table 1:** Similarity index (SI) of DGGE profiles related to T0 and T1

Subject	SI (%)
1	71.8
2	60.6
3	79.2
4	54.1
5	91.3
6	55.9
7	77.5
8	47.7
9	65.0
10	89.3
11	80.9
12	38.2
13	76.1
14	64.7
15	66.6
16	59.4
17	80.3
18	64.3
19	72.1
20	46.4

Cluster analysis of DGGE population profiling confirmed the stability of the overall structure of the microbiome, revealing no grouping according to the feeding (Figure 1B-C). T0 and T1 banding patterns were closely related for all the volunteers, except for the subject 8 (SI: 47.7%). Among different subjects, considerable variation in the composition of the population fingerprints could be observed. Both qualitative (presence or absence of a band) or quantitative (variable intensity of a band) variations did occur. These inter-individual variations were higher than changes elicited by the functional food consumed.

### Quantitative variations of bifidobacteria and lactobacilli

In order to evaluate the effect of the prebiotic component on modulation of bifidobacteria and lactobacilli populations and the capability of the probiotic bacteria to pass through the gut of the healthy host, quantitative variations of *Bifidobacterium *and *Lactobacillus *genera were determined by real-time PCR and compared to the variations of the species *B. longum *and *L. helveticus *(Table 2). All volunteers naturally harbored strains belonging to *Bifidobacterium *and *Lactobacillus*, as demonstrated by the presence of these genera in all stool samples recovered before the beginning of the feeding trial. *B. longum *was also found in all healthy subjects at the time point T0, in accordance with previous studies reporting *B. longum *as one of the major bifidobacterial species in the intestinal microbiota of human adults [29]. Differently, *L. helveticus *was detected only in 8 subjects at the time point T0, showing a frequency of 40%. *L. helveticus *is not a normal inhabitant of the intestinal microbiota, but strains belonging to this species are used as starter cultures in the manufacturing of a variety of fermented dairy products, to modulate flavor. Thus, presence of *L. helveticus *in fecal samples can be related to a diet rich in yogurt and cheese [45]. Table 2 highlights different trends of variation of *Bifidobacterium*, *Lactobacillus*, *B. longum *and *L. helveticus *concentrations among the subjects enrolled in the trial, suggesting a specific individual response to the dietary intervention. This variability is particularly evident for *L. helveticus*. In the majority of the volunteers, the synbiotic intake was associated to an increase or to the appearance of this species. In 2 subjects (4 and 9) no variation was found at the time point T1. In 4 subjects (6, 8, 19 and 20) *L. helveticus *did not appear after the feeding period and in the subject 20 it disappeared at the time point T1. These results indicate that the capability of *L. helveticus *Bar 13 to persist in the gastrointestinal tract is related to the specific characteristics of the host gut environment.

**Table 2:** Real-time PCR quantification of bifidobacteria and lactobacilli

Subject	Time point	16S *rrn *operons/μg fecal genomic DNA (mean ± SD)			
	
		*Bifidobacterium*	*B. longum*	*Lactobacillus*	*L. helveticus*
1	T0	9.4 × 10^6 ^± 3.7 × 10^6^	3.2 × 10^6 ^± 1.5 × 10^6^	2.6 × 10^6 ^± 9.6 × 10^5^	0.0 ± 0.0
	T1	4.1 × 10^6 ^± 8.3 × 10^5^	1.1 × 10^6 ^± 2.9 × 10^5^	1.9 × 10^6 ^± 9.9 × 10^5^	4.5 × 10^2 ^± 2.9 × 10^2^
2	T0	8.9 × 10^7 ^± 3.1 × 10^7^	4.2 × 10^7 ^± 3.6 × 10^7^	1.1 × 10^5 ^± 5.6 × 10^4^	9.0 × 10^1 ^± 6.2 × 10^1^
	T1	1.6 × 10^7 ^± 5.0 × 10^6^	4.7 × 10^6 ^± 2.9 × 10^5^	5.1 × 10^5 ^± 2.4 × 10^5^	2.6 × 10^3 ^± 2.8 × 10^2^
3	T0	4.0 × 10^8 ^± 3.6 × 10^7^	8.6 × 10^6 ^± 2.6 × 10^6^	5.6 × 10^4 ^± 3.5 × 10^4^	0.0 ± 0.0
	T1	2.4 × 10^8 ^± 2.5 × 10^7^	2.4 × 10^7 ^± 2.9 × 10^6^	2.6 × 10^5 ^± 1.6 × 10^5^	2.8 × 10^3 ^± 1.8 × 10^3^
4	T0	2.6 × 10^8 ^± 2.8 × 10^7^	2.3 × 10^7 ^± 2.9 × 10^6^	1.6 × 10^5 ^± 1.0 × 10^3^	2.1 × 10^3 ^± 8.7 × 10^1^
	T1	5.8 × 10^8 ^± 1.2 × 10^7^	3.7 × 10^7 ^± 3.1 × 10^6^	1.2 × 10^5 ^± 2.7 × 10^4^	1.6 × 10^3 ^± 2.2 × 10^2^
5	T0	3.1 × 10^6 ^± 8.6 × 10^5^	9.8 × 10^5 ^± 2.8 × 10^5^	1.9 × 10^4 ^± 5.8 × 10^3^	0.0 ± 0.0
	T1	2.4 × 10^6 ^± 7.3 × 10^5^	9.5 × 10^5 ^± 3.4 × 10^5^	6.1 × 10^4 ^± 3.4 × 10^4^	3.5 × 10^2 ^± 2.3 × 10^2^
6	T0	1.7 × 10^8 ^± 3.8 × 10^7^	6.5 × 10^6 ^± 2.4 × 10^5^	2.7 × 10^5 ^± 1.2 × 10^5^	0.0 ± 0.0
	T1	6.2 × 10^8 ^± 4.2 × 10^7^	3.5 × 10^7 ^± 2.0 × 10^5^	1.7 × 10^5 ^± 1.1 × 10^5^	0.0 ± 0.0
7	T0	6.4 × 10^7 ^± 4.8 × 10^6^	3.4 × 10^7 ^± 1.2 × 10^6^	4.0 × 10^5 ^± 1.7 × 10^5^	9.0 × 10^1 ^± 8.2 × 10^1^
	T1	7.5 × 10^7 ^± 1.2 × 10^6^	4.6 × 10^7 ^± 5.5 × 10^6^	9.2 × 10^5 ^± 4.9 × 10^5^	1.4 × 10^4 ^± 3.2 × 10^3^
8	T0	1.8 × 10^6 ^± 5.8 × 10^5^	6.0 × 10^5 ^± 3.6 × 10^5^	1.0 × 10^6 ^± 1.0 × 10^6^	0.0 ± 0.0
	T1	4.1 × 10^6 ^± 8.5 × 10^5^	1.3 × 10^6 ^± 9.7 × 10^5^	1.7 × 10^5 ^± 1.7 × 10^5^	0.0 ± 0.0
9	T0	4.4 × 10^6 ^± 2.8 × 10^5^	3.0 × 10^6 ^± 2.3 × 10^6^	9.2 × 10^5 ^± 9.0 × 10^5^	3.0 × 10^3 ^± 1.1 × 10^3^
	T1	5.6 × 10^6 ^± 1.4 × 10^5^	3.8 × 10^6 ^± 1.3 × 10^6^	2.0 × 10^6 ^± 1.0 × 10^6^	1.8 × 10^3 ^± 1.7 × 10^3^
10	T0	1.0 × 10^8 ^± 1.8 × 10^7^	7.0 × 10^7 ^± 4.5 × 10^7^	7.7 × 10^5 ^± 7.6 × 10^5^	0.0 ± 0.0
	T1	3.3 × 10^8 ^± 7.7 × 10^7^	4.3 × 10^7 ^± 2.5 × 10^7^	1.3 × 10^6 ^± 1.2 × 10^6^	3.2 × 10^3 ^± 2.7 × 10^3^
11	T0	4.1 × 10^6 ^± 7.5 × 10^5^	1.2 × 10^6 ^± 2.5 × 10^5^	5.1 × 10^5 ^± 4.1 × 10^5^	6.0 × 10^2 ^± 3.8 × 10^2^
	T1	3.4 × 10^7 ^± 6.2 × 10^5^	3.1 × 10^7 ^± 1.0 × 10^7^	7.8 × 10^5 ^± 7.7 × 10^5^	1.7 × 10^4 ^± 3.1 × 10^3^
12	T0	3.4 × 10^5 ^± 7.6 × 10^4^	7.5 × 10^2 ^± 3.0 × 10^1^	1.7 × 10^7 ^± 1.1 × 10^7^	0.0 ± 0.0
	T1	1.3 × 10^6 ^± 7.0 × 10^5^	2.0 × 10^5 ^± 9.3 × 10^4^	5.8 × 10^5 ^± 5.6 × 10^5^	3.6 × 10^3 ^± 6.4 × 10^2^
13	T0	3.5 × 10^7 ^± 1.6 × 10^6^	1.2 × 10^7 ^± 2.6 × 10^5^	1.8 × 10^5 ^± 1.0 × 10^5^	0.0 ± 0.0
	T1	2.3 × 10^7 ^± 3.8 × 10^6^	4.6 × 10^6 ^± 4.4 × 10^5^	2.5 × 10^5 ^± 1.8 × 10^5^	1.8 × 10^2 ^± 4.3 × 10^1^
14	T0	1.1 × 10^7 ^± 6.9 × 10^5^	2.3 × 10^6 ^± 1.6 × 10^6^	1.1 × 10^6 ^± 1.8 × 10^5^	0.0 ± 0.0
	T1	5.4 × 10^7 ^± 1.7 × 10^7^	1.0 × 10^7 ^± 6.5 × 10^6^	7.2 × 10^5 ^± 6.4 × 10^5^	3.0 × 10^2 ^± 3.0 × 10^1^
15	T0	6.1 × 10^7 ^± 7.4 × 10^6^	1.7 × 10^7 ^± 8.3 × 10^6^	3.9 × 10^5 ^± 2.9 × 10^5^	1.8 × 10^1 ^± 1.6 × 10^1^
	T1	2.5 × 10^7 ^± 5.3 × 10^6^	1.0 × 10^7 ^± 5.8 × 10^6^	2.5 × 10^5 ^± 2.2 × 10^5^	3.2 × 10^2 ^± 1.4 × 10^2^
16	T0	1.3 × 10^9 ^± 4.5 × 10^8^	4.0 × 10^7 ^± 1.2 × 10^7^	2.0 × 10^6 ^± 1.1 × 10^6^	0.0 ± 0.0
	T1	1.3 × 10^9 ^± 2.0 × 10^8^	2.2 × 10^7 ^± 3.8 × 10^6^	1.0 × 10^6 ^± 8.2 × 10^5^	8.3 × 10^2 ^± 1.4 × 10^1^
17	T0	1.6 × 10^7 ^± 1.6 × 10^6^	5.0 × 10^6 ^± 3.2 × 10^6^	1.3 × 10^7 ^± 2.9 × 10^6^	1.3 × 10^2 ^± 1.1 × 10^2^
	T1	2.2 × 10^7 ^± 1.9 × 10^6^	4.0 × 10^6 ^± 2.7 × 10^6^	1.5 × 10^7 ^± 2.0 × 10^5^	6.6 × 10^2 ^± 9.5 × 10^1^
18	T0	1.1 × 10^5 ^± 3.1 × 10^6^	1.4 × 10^3 ^± 4.4 × 10^2^	3.1 × 10^7 ^± 2.7 × 10^7^	0.0 ± 0.0
	T1	3.7 × 10^5 ^± 8.9 × 10^4^	1.7 × 10^5 ^± 7.3 × 10^4^	3.0 × 10^6 ^± 1.2 × 10^6^	6.5 × 10^2 ^± 1.2 × 10^2^
19	T0	5.2 × 10^7 ^± 1.7 × 10^7^	4.3 × 10^5 ^± 1.8 × 10^5^	2.5 × 10^6 ^± 1.9 × 10^6^	0.0 ± 0.0
	T1	2.0 × 10^7 ^± 8.0 × 10^6^	1.5 × 10^5 ^± 9.4 × 10^4^	2.0 × 10^6 ^± 1.5 × 10^6^	0.0 ± 0.0
20	T0	6.6 × 10^6 ^± 5.2 × 10^6^	4.4 × 10^6 ^± 2.2 × 10^6^	1.0 × 10^7 ^± 8.4 × 10^6^	1.8 × 10^3 ^± 2.6 × 10^2^
	T1	7.0 × 10^6 ^± 3.3 × 10^5^	5.5 × 10^6 ^± 3.3 × 10^6^	2.7 × 10^5 ^± 2.6 × 10^5^	0.0 ± 0.0

**Figure 2:**
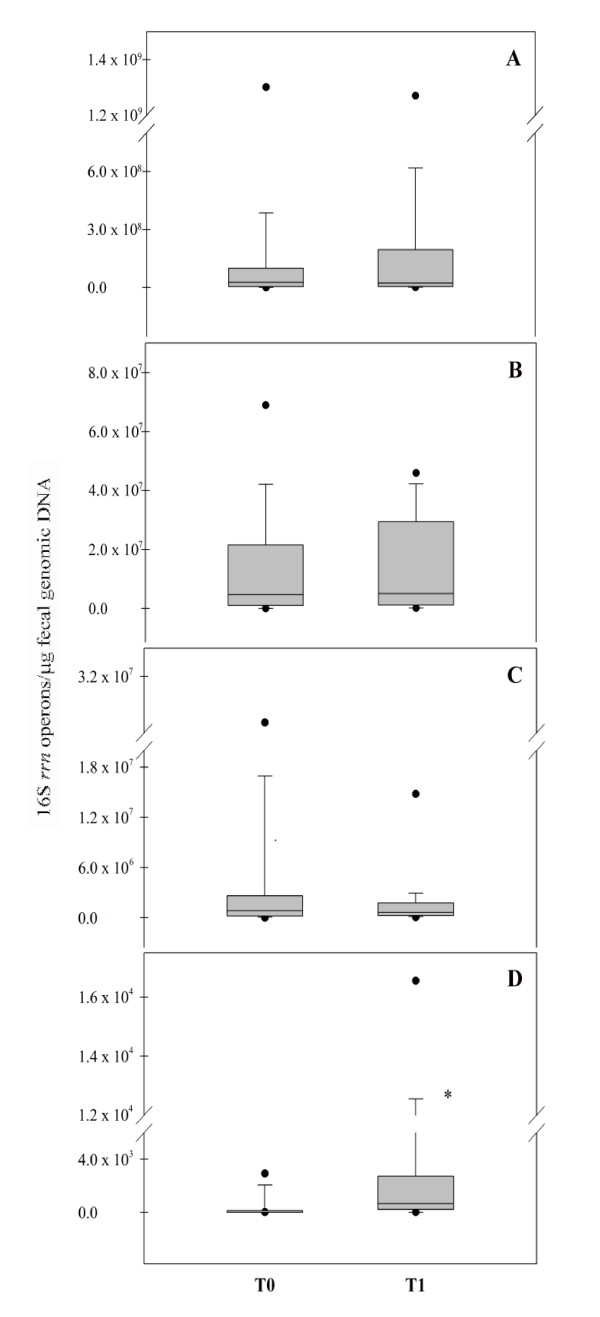
**Real-time PCR evaluation of 16S *rrn *operons of *Bifidobacterium *(A), *B. longum *(B), *Lactobacillus *(C) and *L. helveticus *(D) related to the time points (T0 and T1) of the feeding study**. Data are expressed as number of operons in 1 μg of total bacterial DNA extracted from the feces. The box represents the interquartile range (25-75th percentile) and the line within the box is the median value. The bottom and top bars indicate the 10th and 90th percentiles, respectively. Outlier values are indicated (black circles). * indicates a significant difference (*P *< 0.05).

In order to assess the global impact of the functional food consumption on the bifidobacteria and lactobacilli populations, a statistical elaboration of the real-time PCR data was performed. Box plots in Figure 2 show the amounts of 16S *rrn *operons of *Bifidobacterium *(A), *B. longum *(B), *Lactobacillus *(C) and *L. helveticus *(D) detected at the time points T0 and T1 of the feeding study. The intake of the synbiotic food did not cause significant variations in the median value of *Bifidobacterium *(T0: 2.6 × 10^7^; T1: 2.2 × 10^7^), *B. longum *(T0: 4.7 × 10^6^; T1: 5.1 × 10^6^) and *Lactobacillus *(T0: 8.5 × 10^5^; T1: 6.5 × 10^5^). On the contrary, a significant increase (*P *< 0.05) of *L. helveticus *DNA was observed after the administration of the functional food (T0 median value: 0; T1 median value: 6.6 × 10^2^), demonstrating the ability of *L. helveticus *Bar13 to pass through the gut of healthy humans. The significant increase of *L. helveticus *was directly linked to the low incidence of this species in the intestine of the human host. Analogously, the absence of significant variations in *Bifidobacterium*, *Lactobacillus *and *B. longum *could be related to the high T0 amounts of these bacterial groups, natural inhabitants of the gut microbiota of healthy humans. Amounts of *L. helveticus *were evaluated by real-time PCR in stool samples recovered from 10 subjects after a wash-out period of 20 days. Concentration of this species returned to a median value of 0, supporting the hypothesis of a transient persistence of the probiotic strain Bar13 during the feeding period (data not shown).

**Figure 3:**
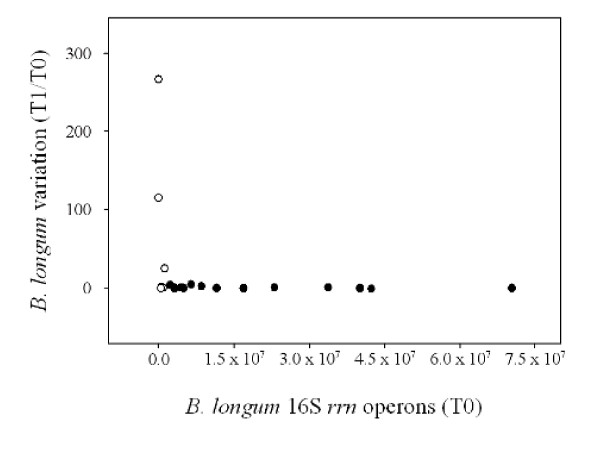
**Relationship between *B. longum *variations (T1/T0 16S *rrn *operons) and *B. longum *amount before the feeding trial (T0 16S *rrn *operons)**. Empty circles indicate subjects with T0 value minor or equal to 1.0 × 10^6 ^16S *rrn *operons per μg of total bacterial DNA. Filled circles indicate subjects with T0 value higher than 1.0 × 10^6 ^16S *rrn *operons per μg of total bacterial DNA.

Figure 3 shows the relationship between the variation of *B. longum *species, expressed as the ratio of T1 and T0 16S *rrn *operons, and the basal concentration of *B. longum*, expressed as the number of 16S *rrn *operons measured at the time point T0. The trend of the curve indicates a strong influence of the initial concentration of *B. longum *on the variation of *B. longum *population after the feeding period. An evident increase of *B. longum *was observed in subjects 11, 12 and 18, who showed T0 amount of this species minor or equal to 1.0 × 10^6 ^16S *rrn *operons per μg of total bacterial DNA. Notably, subject 12, presenting the lowest *B. longum *concentration at the time point T0 (7.5 × 10^2^), showed the highest variation of *B. longum *(T1/T0: 2.6 × 10^2^) after the synbiotic intake. The same subject presented the lowest SI (38.2%) between DGGE band profiles related to the time points T0 and T1. These data suggest the capability of *B. longum *Bar33 to pass through the human gastrointestinal tract, but this property can be detected only in subjects harboring low basal level of *B. longum *species.

### Changes in intestinal metabolic profiles

**Figure 4:**
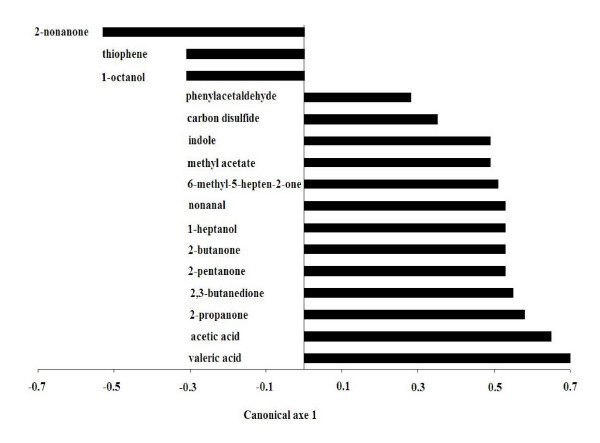
**CAP loadings plot of metabolites whose concentration was significantly affected by the intake of the synbiotic food (*P *< 0.05)**. Positive and negative coefficients indicate the increase or decrease of metabolite amounts following the feeding period.

**Figure 5:**
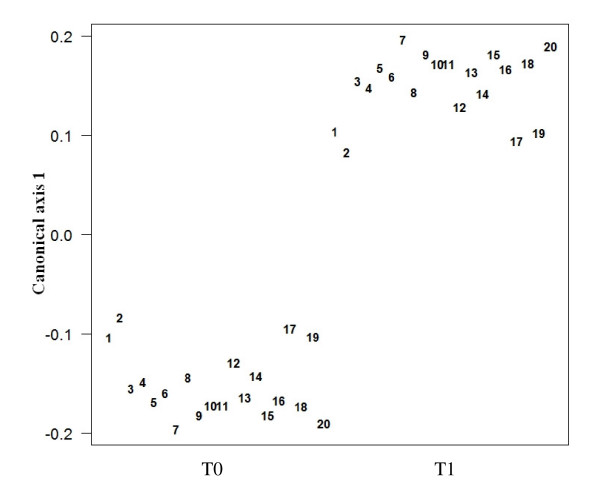
**CAP scores plot of the stool samples collected from the twenty volunteers before (T0) and after (T1) the synbiotic food intake**.

In this investigation about 130 different metabolites belonging to the families of alcohols, ketones, aldehydes, sulfur compounds, nitrogen compounds and SCFA were detected in feces by means of GC-MS/SPME analysis (see Additional file 1). A two-tailed Mann-Whitney test was performed on the metabolic data matrix in order to identify the molecules significantly affected by the consumption of the functional food. A CAP analysis performed on the selected molecules evidenced that metabolites whose changes were positively correlated with the synbiotic administration principally belonged to the families of ketones (methyl-5-hepten-2-one, 2-propanone, 2-butanone, 2-pentanone, 2,3-butanedione) and SCFA (acetic and valeric acid). Differently, the concentration of 1-octanol, thiophene and nonanone decreased significantly after the feeding period. These results are showed in the Figure 4, which reports the loadings plot obtained from the CAP analysis. The scores plot (canonical axe) obtained from the same supervised method showed a perfect classification of the samples, on the basis of the synbiotic food intake (Figure 5). The application of the CAP analysis on metabolites data set characterized by GC-MS/SPME resulted in classification and predictive abilities of 100% (see Additional file 2), as evaluated by the leave-four-out procedure used, using only a reduced number of experimental chromatographic peaks as input variables.

## Discussion

The significant involvement of the gut microbiota in the human health suggests that modulation of commensal microbial composition and metabolism through combinations of probiotics and prebiotics could be a dietary strategy to prevent diverse diseases, such as obesity, diabetes, non-alcoholic fatty liver disease, inflammatory bowel disease, and even cancers [4].

In the present study, the impact of a synbiotic food supplement on the gut microbiota structure of healthy humans was evaluated by using an integrated molecular approach based on PCR-DGGE and real-time PCR.

DGGE profiles of the predominant fecal microbiota generated complex but overall relatively stable and unique profiles for each individual. Elaboration of DGGE data revealed high SI values between T0 and T1 profiles, and no clustering of banding patterns according to the feeding. These results demonstrated that no significant change in the structure of the gut microbiota of healthy subjects did occur following dietary intervention, confirming previous findings regarding the subject specificity of the predominant fecal communities and their stability over time and resistance to perturbations [9,23]. Notably, we cannot exclude an effect of the synbiotic intake on minor bacterial species, an effect that could be investigated using high-throughput sequencing techniques. However, the impact on the dominant colonic microbiota represents the main parameter to evaluate the clinical relevance for the use of a functional food.

Because DGGE can be considered a semiquantitative tool for monitoring the dynamics of the predominant bacterial species of an ecosystem, additional analysis with real-time PCR was performed to obtain a quantitative estimation of the effect of the synbiotic intake on bifidobacteria and lactobacilli populations. In particular, variations in amounts of *B. longum *and *L. helveticus *were evaluated in order to assess the capability of the probiotic species included in the synbiotic food to pass through the gastrointestinal tract of the human host. Only *L. helveticus *concentration increased significantly after the ingestion of the functional food, demonstrating the gut persistence of the probiotic *L. helveticus *strain during the feeding period. Since *L. helveticus *species is not a natural inhabitant of the human intestine and its presence in feces is diet related [45], this result was not surprising and suggests that low abundant species could be optimal models for studying the gut colonization of probiotic bacteria. On the other hand, visualization of the gut colonization of a high abundant species, such as *B. longum*, is strictly related to its basal concentration. For this reason, we observed the *B. longum *increase only in subjects with the lowest concentration of *B. longum *species at the time point T0.

The intake of the synbiotic food resulted in significant changes in some gut metabolic activities, as highlighted by the CAP analysis of the fecal metabolic profiles, which pointed out a separation of fecal samples of the subjects on the basis of the synbiotic food intake. Surprisingly little is known about volatile organic compounds formed in the gut. GC-MS/SPME, detecting volatile molecules with high sensitivity, represents a suitable approach to identify microbial metabolites in fecal samples, such as SCFAs, ketones, esters and sulfur compounds [46].

Two SCFAs, acetic and valeric acids, were the metabolites showing the highest increase after the synbiotic administration. Although a general increase was observed also for butyric acid, this variation was not statistically significant due to the high variability of the measures. SCFAs are very common in the gut environment, arising from metabolism of undigested carbohydrates, such as dietary fiber and prebiotics, by colonic bacteria. The increase of SCFAs is particularly interesting, as they play a role in regulation of cell proliferation and differentiation of the colonic epithelial cells. Increases in SCFA production have been associated with decreased pH, which may reduce potential pathogenic clostridia, decreased solubility of bile acids, increased absorption of minerals, and reduced ammonia absorption by the protonic dissociation of ammonia and other amines [47]. Other metabolites whose changes were positively and significantly correlated with the synbiotic intake belonged to the family of methylketones (methyl-5-hepten-2-one, 2-propanone, 2-butanone, 2-pentanone, 2,3-butanedione). In particular, the significant increase of 2-pentanone can be regarded as the most interesting effect associated with the synbiotic food intake. In fact, 2-pentanone, which is a naturally occurring compound in fruits, vegetables and fermented foods, has anti-inflammatory and chemopreventive properties. According to Pettersson *et al*. [48], it inhibits the prostaglandin production and COX-2 protein expression in human colon cancer cells. The increase of 2,3-butanedione is interesting since it may have health benefits by impacting on the growth of some bacteria, such as *L. delbrueckii *subsp. *bulgaricus *ad *Streptococcus thermophilus *[41]. Furthermore, during glucose catabolism 2,3-butanedione serves as an electron acceptor and can be reduced to 2,3-butanediol via acetoin. This pathway was shown to be important in the removal of toxic amounts of pyruvate and in maintenance of pH homeostasis [49]. A diverse range of sulfur compounds has been identified in stool samples [41]. The usual source of sulfur compounds is the microbial breakdown of sulfur containing amino acids and the increase of these compounds suggests an abundance or metabolic activity of bacteria able to breakdown cystein and methionine. In our study, a significant increase of carbon disulfide was observed following the feeding period. Carbon disulfide may be produced by carbonation of hydrogen sulphide as a detoxification mechanism exerted by colonic bacteria. According to Garner *et al*. [41], carbon disulfide has been found in 100% of the samples from healthy donors and absent in many samples of patients with *Campylobacter jejuni *and *Clostridium difficile*. Various esters were detected in all fecal samples. In particular, a significant increase of methyl acetate, ester of methanol and acetic acid, was evident after the synbiotic intake. Methanol is rarely found as free alcohol in the gut, where it is generated from the breakdown of macromolecules including pectins, bran and aspartame. In general, free alcohols and endogenous fatty acids are metabolized into fatty acid esters in liver, pancreas and intestine [50]. At the intestinal site, esterification of alcohols by colonic bacteria can be regarded as a microbial strategy to remove or trap toxic molecules such as fatty acids and alcohols.

To sum up, the investigation of the fecal volatile metabolites by GC-MS/SPME allowed to correlate the consumption of the synbiotic food with the stimulation of health-promoting metabolic activities of the gut microbiota, such as regulation of the colonic epithelial cell proliferation and differentiation, anti-inflammatory and chemopreventive properties and detoxification processes.

## Conclusion

In the current study molecular fingerprinting techniques (PCR-DGGE and real-time PCR) were integrated to the GC-MS/SPME analysis of the metabolic profiles to investigate the global impact of a dietary intervention on the gut ecology and metabolism in healthy humans. In particular, the major findings of this study are the following: (i) the synbiotic food does not modify the overall structure of the gut microbiome, as detected by DGGE; (ii) the gut survival of the probiotic strains may be supposed on the basis of the increase of *B. longum *and *L. helveticus *after the synbiotic consumption; (iii) the perturbation of the gut metabolism triggered by a synbiotic food intake generates significant changes in the GC-MS/SPME profiles; (iv) changes in metabolic phenotypes seem to indicate potential implications of the synbiotic food in health maintenance and prevention of diverse diseases.

In order to better investigate the mechanistic basis of the probiotics and prebiotics action on gut microbial activities and the outcomes on human health, it will be necessary to integrate the GC-MS/SPME and/or NMR profiles of feces with simultaneous analysis of different biofluids, including urine and plasma.

## Methods

### Study population

Twenty randomly selected healthy volunteers (11 women and 9 men) aged between 20 and 50 (mean: 35) participated in the study. The Ethics Committee of the University of Bologna (Italy) approved the study, and all subjects gave informed consent. None of the subjects had a history of gastrointestinal or metabolic disease or previous surgery (apart from appendectomy). The subjects did not receive antibiotic treatment or any other medical treatment influencing intestinal microbiota during 3 months before the start of the study. Subjects maintained their usual diet during the study period. All the volunteers had normal weight with a body mass index in the range 18.5-24.9. The volunteers received one dose of a synbiotic snack (Barilla, Parma, Italy), twice a day for a period of 1 month. The synbiotic bar consisted of a biscuit covered by chocolate. The biscuit contained 500 mg of FOS (Actilight^® ^950P, Marckolsheim, France) and the chocolate included a mixture of the probiotic strains *B. longum *Bar33 and *L. helveticus *Bar13 (Barilla culture collection). 10^9^CFU of each probiotic strain were present in a dose of the synbiotic bar.

### Extraction of DNA from fecal samples

Stool samples were collected from volunteers before the start of the feeding study (T0) and at the end of the ingestion period (T1) and immediately frozen at -80°C until use. Total DNA was extracted from 230 mg of feces by using QIAamp DNA Stool Mini Kit (Qiagen, Hilden, Germany), according to the manufacturer's instructions.

### PCR-DGGE and cluster analysis

Amplification of the V2-V3 region of the bacterial 16S rRNA gene was carried out using the universal eubacterial primers GCclamp-HDA1 and HDA2 [51], supplied by M-Medical (Milan, Italy). The amplification reactions were performed in a Biometra Thermal Cycler T Gradient (Biometra, Göttingen, Germany). AmpliTaq Gold DNA Polymerase (Applied Biosystem, Foster City, CA) was used as thermostable DNA polymerase. The reaction mixture contained 0.5 μM of each primer, 200 μM of each dNTP, 0.5 U of DNA Polymerase, and 4 μl of the bacterial DNA template in a final volume of 50 μl. The thermocycle program consisted of the following time and temperature profile: 95°C for 15 min; 30 cycles of 95°C for 60 s, 56°C for 30 s, 72°C for 30 s; and 72°C for 8 min. A volume of 15-20 μl of PCR samples was used for DGGE analysis, which was performed by using the D-Code Universal Mutation System Apparatus (Bio-Rad, Los Angeles, CA), as previously described [52]. Briefly, the sequence-specific separation of the PCR fragments was obtained in 8% (w/v) polyacrylamide gels, containing a 30% to 50% gradient of urea and formamide. Electrophoresis was started at a voltage of 250 V for 5 minutes and continued at constant voltage of 90 V and temperature of 60°C for 16 h. Following electrophoresis, the gel was silver stained [53] and scanned using a Molecular Imager Gel Doc XR System (Bio-Rad). DGGE gel images were analyzed using the FPQuest Software Version 4.5 (Bio-Rad). In order to compensate for gel-to-gel differences and external distortion to electrophoresis, the DGGE patterns were aligned and normalized using an external reference ladder, containing PCR amplicons from pure cultures of intestinal bacterial species. A cluster analysis of the DGGE patterns was performed using the FPQuest Software. The similarity in the profiles was calculated on the basis of the Pearson correlation coefficient with the Ward clustering algorithm.

### Development of *L. helveticus *species-specific primers

By using 16S and 16S-23S rRNA sequences obtained from the DDBJ and EMBL databases, multiple alignments of sequences related to *L. helveticus *and reference organisms were constructed with the program Clustal W http://www.ebi.ac.uk/Tools/clustalw2. Potential target sites for specific detection of the species *L. helveticus *were identified and the following primers were designed: F_Hel (5'-GTGCCATCCTAAGAGATTAGGA-3') and R_Hel (5'-TATCTCTACTCTCCATCACTTC-3'). A Blast search http://www.ncbi.nlm.nih.gov/BLAST was carried out to test the virtual specificity of the primers. Validation of specificity was performed by PCR experiments against different species of *Lactobacillus *(*L. acidophilus*, *L. casei*, *L. plantarum*, *L. bulgaricus*, *L. reuteri, L. gasseri, L. johnsonii*) and other intestinal genera (*Bifidobacterium*, *Streptococcus*, *Escherichia*). The primers were synthesized by M-Medical (Milan, Italy) and optimal annealing temperature was established by gradient PCR.

### Real-time quantitative PCR

Quantitative PCR was performed in a LightCycler instrument (Roche, Mannheim, Germany) and SYBR Green I fluorophore was used to correlate the amount of PCR product with the fluorescence signal. The following genus- and species-specific primers sets, targeted to 16S or 16S-23S rRNA sequences, were used: Bif164/Bif662 (*Bifidobacterium *[54]); Lac1/Lab0677r (*Lactobacillus *[55,56]); BiLON1/BiLON2 (*B. longum *[29]); F_Hel/R_Hel (*L. helveticus *[this work]). Three sub-samples of each DNA extract were amplified in a final volume of 20 μl containing 4 mM of MgCl_2_, 0.5 μM of each primer, 2 μl of LightCycler-FastStart DNA Master SYBR Green I (Roche), and either 2 μl of template or water (no-template control). The thermal cycling conditions were as follows: an initial denaturation step at 95°C for 10 min followed by 40 cycles of denaturation at 95°C for 15 s; primer annealing at 60°C (*Bifidobacterium*), 65°C (*Lactobacillus *and *B. longum*) and 63°C (*L. helveticus*) for 25 s; extension at 72°C for 25 s (*Bifidobacterium*), 20 s (*Lactobacillus*), 45 s (*B. longum*) and 10 s (*L. helveticus*) and a fluorescence acquisition step at 90°C (*Bifidobacterium *and *B. longum*) or 85°C (*Lactobacillus *and *L. helveticus*) for 5 s. For each step the temperature transition rate was 20°C/s. Quantification of *rrn *operons of *Bifidobacterium*, *Lactobacillus *and *B. longum *was done by using standard curves made from known concentrations of genomic DNA from the sequenced strains *B. longum *NCC2705 [30] and *L. acidophilus *NCFM [57]. For *L. helveticus *species the probiotic strain included in the synbiotic was used as standard and the number of *rrn *operons in the genome was deduced from the sequenced genome of *L. helveticus *DPC 4571 [58]. Chromosomal DNA of the strains used as standards was extracted by using DNeasy Tissue Kit (Qiagen) and serially diluted from 10^5 ^to 10^1 ^molecules/μl. Results obtained by PCR were converted to the average estimate of total *rrn *operons from each group present in 1 μg of total DNA, and standard deviations (SD) were calculated.

### GC-MS/SPME

A carboxen-polydimethylsiloxane coated fiber (85 μm) and a manual SPME holder (Supelco, Bellefonte, PA) were used in this study after preconditioning according to the manufacturer's instruction manual. Before each head space sampling, the fiber was exposed to the GC inlet for 5 min for thermal desorption at 250°C in a blank sample. Five ml of fecal slurries (20%) were placed in 10 ml glass vials, added with 4-methyl-2-pentanol (4 mg/l) as internal standard. The samples were then equilibrated for 10 min at 45°C. The SPME fiber was exposed to each sample for 40 min and then was inserted into the injection port of the GC for a 5 min sample desorption. GC-MS analyses were performed on an Agilent 7890A gaschromatograph (Agilent Technologies, Palo Alto, CA) coupled to an Agilent 5975C mass selective detector operating in electron impact mode (ionization voltage 70 eV). A Supelcowax 10 capillary column (60 m length, 0.32 mm ID) was used (Supelco). The temperature program was: 50°C for 1 min, then programmed at 4.5°C/min to 65°C and finally at 10°C/min to 230°C which was maintained for 25 min. Injector, interface and ion source temperatures were 250, 250 and 230°C, respectively. The mass-to-charge ratio interval was 30-350 Da at 2.9 scans per second. Injections were performed in splitless mode and helium (1 ml/min) was used as carrier gas. The identification of all the molecules detected in fecal samples was based on comparison of their retention times and spectral data with those of pure compounds (Sigma-Aldrich, Milan, Italy) analyzed in the same conditions. The identification was further confirmed by comparing mass spectra of all compounds with those contained in available databases (NIST version 2005 and Wiley version 1996) and in literature [41]. Quantitative data of the identified compounds were obtained by interpolation of the relative areas versus the internal standard area, in calibration curves built with pure reference compounds. The concentration of volatile compounds, for which there were no pure references, was obtained by using the same calibration graphs of the compounds with the most similar chemical structure.

### Statistical analyses

For each subject, variations of the DGGE profiles related to the time points T0 and T1 were analyzed by Pearson correlation. Significant differences in the intensity of each DGGE band among all fecal samples were searched by using Mann-Whitney U-test. Mann-Whitney U-test was also used to analyze differences in total *rrn *operons of target genera and species and to determine metabolites significantly affected by the synbiotic food intake. A *P *value below 0.05 was considered statistically significant. Metabolites with a *P *value below 0.05 were then used in further multivariate analysis. These selected metabolites formed a matrix containing two kinds of information: the effects of the synbiotic food intake (within-individual variability) and the natural differences between individuals (between-individuals variability). These two kinds of information were separated following the method of Jansen *et al*. [59]. A CAP analysis was then performed on the within-individual variability matrix [60]. The CAP constrained ordination procedure can be summarized as follows: the data were reduced by performing a principal coordinate analysis (PCO) on the parameters using a dissimilarity measure based on Euclidean distances; an appropriate number of PCOs were chosen non-arbitrarily, which maximize the number of observations correctly classified [61,60]. The robustness of the model obtained was established by a 4-fold cross validation method, repeatedly leaving out a fourth of the samples and predicting them back into the model [62]. Finally a traditional canonical analysis on the first three PCOs was performed. The hypothesis of no significant difference in multivariate location among the groups was tested by using a permutation test based on 9999 permutations.

Statistical analyses were performed using the software SigmaStat (Systat Sofware Inc., San Jose, CA) and the package Canoco for Windows 4.5 (Microcomputer Power, Ithaca, NY).

## Authors' contributions

BV performed the study design, analysis and interpretation of the data and the writing of the paper. FC and MC performed the DGGE and real time experiments and statistical analysis of the data. MN carried out GC-MS/SPME experiments. PC, MEG and PB coordinated the study. All authors read and approved the manuscript.

## Supplementary Material

Additional file 1 **Metabolites detected by GC-MS/SPME analysis**. Metabolites were identified and quantified (mg/kg) in stool samples collected from 20 volunteers before (T0) and after (T1) the synbiotic food intake. Click here for file

Additional file 2 **Confusion matrix**. Confusion matrix derived by 4-fold cross-validation of CAP model obtained using metabolites identified in stool samples collected from 20 volunteers before (T0) and after (T1) the synbiotic food intake. Click here for file
