# Supplementary material for: Impact of a synbiotic food on the gut microbial ecology and metabolic profiles
Source: BMC Microbiol. 2010 Jan 7;10:4. doi: 10.1186/1471-2180-10-4 (PMC2806344; doi:10.1186/1471-2180-10-4)
Supplement: Additional file 1 — Metabolites detected by GC-MS/SPME analysis. Metabolites were identified and quantified (mg/kg) in stool samples collected from 20 volunteers before (T0) and after (T1) the synbiotic food intake. [file 1471-2180-10-4-S1.DOC]

**Additional file 1. Metabolites detected by GC-MS/SPME analysis.**

| **Subject** | **Time** | **acetaldehyde** | **carbon disulfide** | **2-propanone** | **methyl acetate** | **2-butanone** | **3-methylbutanal** | **methyl propionate** | **ethanol** |
| --- | --- | --- | --- | --- | --- | --- | --- | --- | --- |
| **1** | T0 | 0.00 | 0.00 | 7.43 | 8.93 | 7.30 | 1.56 | 0.00 | 18.05 |
| **2** | T0 | 4.35 | 2.41 | 16.14 | 3.90 | 10.54 | 1.95 | 0.00 | 4.93 |
| **3** | T0 | 2.00 | 3.51 | 6.66 | 6.78 | 8.35 | 0.00 | 0.00 | 13.65 |
| **4** | T0 | 4.34 | 2.20 | 9.16 | 3.97 | 12.14 | 0.00 | 1.51 | 13.04 |
| **5** | T0 | 1.90 | 5.66 | 7.81 | 7.13 | 15.35 | 0.00 | 4.63 | 3.00 |
| **6** | T0 | 1.57 | 31.28 | 7.32 | 2.88 | 13.05 | 0.00 | 1.78 | 8.38 |
| **7** | T0 | 0.76 | 3.68 | 8.09 | 2.80 | 8.11 | 0.00 | 1.76 | 6.64 |
| **8** | T0 | 3.87 | 2.60 | 29.22 | 0.66 | 24.57 | 0.86 | 0.47 | 24.43 |
| **9** | T0 | 11.21 | 2.48 | 6.83 | 0.76 | 2.46 | 0.13 | 0.18 | 34.11 |
| **10** | T0 | 2.03 | 0.90 | 9.36 | 1.89 | 5.45 | 0.00 | 3.84 | 6.43 |
| **11** | T0 | 3.93 | 4.38 | 6.34 | 6.03 | 4.27 | 1.83 | 0.00 | 29.01 |
| **12** | T0 | 1.80 | 3.19 | 12.32 | 3.55 | 8.82 | 1.06 | 0.01 | 20.79 |
| **13** | T0 | 8.59 | 3.22 | 16.35 | 6.02 | 10.30 | 0.00 | 2.76 | 3.97 |
| **14** | T0 | 0.98 | 2.69 | 11.29 | 1.47 | 6.95 | 1.05 | 0.00 | 2.14 |
| **15** | T0 | 2.55 | 1.15 | 9.96 | 0.28 | 4.22 | 0.40 | 0.42 | 9.12 |
| **16** | T0 | 4.69 | 2.65 | 8.29 | 3.15 | 6.34 | 0.00 | 1.36 | 5.18 |
| **17** | T0 | 17.75 | 2.73 | 7.99 | 2.84 | 5.52 | 0.00 | 1.43 | 8.41 |
| **18** | T0 | 2.19 | 4.52 | 19.26 | 5.53 | 16.05 | 0.53 | 0.00 | 18.71 |
| **19** | T0 | 3.44 | 2.98 | 6.93 | 7.43 | 5.69 | 0.40 | 0.00 | 78.28 |
| **20** | T0 | 6.82 | 2.51 | 18.44 | 2.17 | 14.13 | 0.00 | 0.00 | 13.48 |
| **1** | T1 | 5.93 | 0.00 | 9.05 | 2.54 | 6.28 | 1.04 | 0.00 | 7.95 |
| **2** | T1 | 11.59 | 1.98 | 13.05 | 9.14 | 10.86 | 0.00 | 1.93 | 12.73 |
| **3** | T1 | 4.75 | 1.07 | 6.20 | 2.05 | 3.30 | 0.00 | 0.00 | 8.02 |
| **4** | T1 | 7.99 | 0.00 | 10.71 | 6.60 | 11.37 | 0.00 | 0.00 | 23.68 |
| **5** | T1 | 3.17 | 7.82 | 6.91 | 2.18 | 8.29 | 0.00 | 2.25 | 10.32 |
| **6** | T1 | 4.42 | 3.76 | 9.50 | 4.17 | 8.58 | 0.00 | 2.89 | 11.56 |
| **7** | T1 | 3.26 | 4.38 | 6.42 | 2.93 | 6.57 | 0.00 | 2.68 | 8.12 |
| **8** | T1 | 8.26 | 4.95 | 17.10 | 0.00 | 6.66 | 0.55 | 0.00 | 21.22 |
| **9** | T1 | 6.62 | 5.22 | 9.24 | 4.92 | 8.11 | 0.51 | 1.49 | 28.33 |
| **10** | T1 | 4.98 | 3.39 | 12.10 | 0.00 | 4.33 | 0.00 | 2.59 | 10.70 |
| **11** | T1 | 3.39 | 3.04 | 8.65 | 5.48 | 6.92 | 0.96 | 0.35 | 27.98 |
| **12** | T1 | 23.59 | 2.12 | 6.72 | 5.81 | 7.91 | 2.42 | 4.01 | 17.83 |
| **13** | T1 | 5.21 | 2.14 | 7.58 | 2.12 | 5.12 | 1.95 | 0.00 | 10.75 |
| **14** | T1 | 8.09 | 3.10 | 6.13 | 0.00 | 5.42 | 1.00 | 0.00 | 4.72 |
| **15** | T1 | 7.16 | 0.00 | 9.73 | 0.00 | 5.89 | 0.92 | 1.24 | 17.58 |
| **16** | T1 | 9.63 | 0.00 | 8.23 | 4.09 | 5.17 | 1.14 | 1.95 | 10.70 |
| **17** | T1 | 6.54 | 1.64 | 6.50 | 1.88 | 5.33 | 0.00 | 2.76 | 9.63 |
| **18** | T1 | 2.16 | 2.77 | 5.90 | 4.47 | 32.40 | 0.40 | 0.00 | 15.77 |
| **19** | T1 | 3.59 | 10.00 | 16.28 | 11.54 | 24.77 | 0.70 | 0.00 | 55.11 |
| **20** | T1 | 7.75 | 1.70 | 5.94 | 2.61 | 6.05 | 2.54 | 1.60 | 27.92 |

| **Subject** | **Time** | **2,3-butanedione** | **2-pentanone** | **ethyl butyrate** | **methyl isovalerate** | **ethyl isovalerate** | **ethyl valerate** | **1-butanol** | **butyl butanoate** |
| --- | --- | --- | --- | --- | --- | --- | --- | --- | --- |
| **1** | T0 | 9.71 | 25.88 | 15.57 | 0.00 | 0.00 | 0 | 72.55 | 1.31 |
| **2** | T0 | 10.28 | 10.85 | 3.19 | 1.22 | 0.00 | 0 | 37.40 | 0.00 |
| **3** | T0 | 4.80 | 12.53 | 0.88 | 0.00 | 0.00 | 0 | 57.31 | 12.28 |
| **4** | T0 | 8.63 | 14.14 | 1.91 | 0.00 | 10.09 | 0 | 43.65 | 8.30 |
| **5** | T0 | 9.76 | 46.68 | 2.24 | 8.31 | 4.77 | 0 | 30.89 | 10.70 |
| **6** | T0 | 3.47 | 24.82 | 0.00 | 3.81 | 4.56 | 0 | 27.07 | 1.51 |
| **7** | T0 | 6.92 | 11.50 | 0.53 | 7.68 | 4.53 | 0 | 46.64 | 1.35 |
| **8** | T0 | 9.30 | 6.23 | 0.87 | 5.18 | 0.00 | 0.00 | 0.44 | 32.65 |
| **9** | T0 | 39.67 | 3.02 | 0.46 | 21.48 | 6.43 | 0.19 | 20.84 | 7.35 |
| **10** | T0 | 21.19 | 9.33 | 0.00 | 16.81 | 33.04 | 0 | 0.48 | 34.31 |
| **11** | T0 | 19.01 | 7.83 | 0.00 | 5.73 | 43.17 | 0.00 | 34.28 | 7.88 |
| **12** | T0 | 10.69 | 5.76 | 7.01 | 4.23 | 18.63 | 0.27 | 25.63 | 15.58 |
| **13** | T0 | 4.74 | 29.67 | 0.00 | 8.12 | 19.54 | 0 | 0.00 | 46.51 |
| **14** | T0 | 35.59 | 5.11 | 0.00 | 30.93 | 0.00 | 0 | 0.00 | 19.54 |
| **15** | T0 | 22.68 | 2.10 | 0.00 | 0.00 | 17.17 | 0 | 11.51 | 5.80 |
| **16** | T0 | 9.05 | 8.06 | 0.00 | 11.99 | 5.07 | 0 | 0.00 | 17.37 |
| **17** | T0 | 8.97 | 4.63 | 0.00 | 27.63 | 6.61 | 0 | 4.26 | 29.11 |
| **18** | T0 | 36.79 | 11.14 | 1.44 | 11.22 | 51.96 | 0.08 | 25.60 | 6.12 |
| **19** | T0 | 42.93 | 4.25 | 5.61 | 11.45 | 143.42 | 0.88 | 54.00 | 1.78 |
| **20** | T0 | 5.36 | 13.16 | 0.00 | 10.00 | 6.31 | 0 | 0.00 | 29.35 |
| **1** | T1 | 10.95 | 7.19 | 3.14 | 0.00 | 0.00 | 0 | 31.68 | 3.91 |
| **2** | T1 | 9.78 | 24.65 | 14.66 | 9.06 | 0.00 | 0 | 59.92 | 4.79 |
| **3** | T1 | 6.26 | 3.65 | 0.00 | 0.00 | 0.00 | 0 | 22.79 | 7.23 |
| **4** | T1 | 3.21 | 9.13 | 6.50 | 0.00 | 0.00 | 0 | 43.43 | 8.40 |
| **5** | T1 | 10.25 | 32.37 | 6.67 | 9.77 | 2.89 | 0 | 25.62 | 9.44 |
| **6** | T1 | 4.40 | 8.79 | 0.00 | 11.28 | 9.64 | 0 | 27.08 | 7.95 |
| **7** | T1 | 7.15 | 26.26 | 0.61 | 3.99 | 12.91 | 0 | 39.77 | 2.61 |
| **8** | T1 | 14.15 | 8.58 | 5.90 | 10.06 | 36.07 | 17.75 | 33.20 | 14.63 |
| **9** | T1 | 30.19 | 5.73 | 0.07 | 5.86 | 5.24 | 0.14 | 50.70 | 23.76 |
| **10** | T1 | 5.99 | 4.03 | 0.00 | 10.21 | 8.76 | 0 | 0.00 | 15.48 |
| **11** | T1 | 14.51 | 10.50 | 4.61 | 4.11 | 1.00 | 0.54 | 66.54 | 12.24 |
| **12** | T1 | 13.77 | 21.20 | 2.18 | 25.25 | 9.44 | 0.73 | 49.75 | 11.92 |
| **13** | T1 | 7.02 | 7.94 | 0.00 | 10.50 | 3.66 | 0 | 14.53 | 2.00 |
| **14** | T1 | 14.94 | 5.87 | 0.00 | 17.56 | 5.93 | 0 | 20.79 | 3.00 |
| **15** | T1 | 2.48 | 7.92 | 0.00 | 11.32 | 12.41 | 0 | 29.39 | 3.40 |
| **16** | T1 | 5.47 | 13.83 | 0.00 | 14.52 | 12.46 | 0 | 41.25 | 3.20 |
| **17** | T1 | 8.95 | 4.45 | 0.00 | 33.41 | 17.16 | 0 | 20.38 | 2.90 |
| **18** | T1 | 0.90 | 5.15 | 38.77 | 1.23 | 35.84 | 0.98 | 25.95 | 2.16 |
| **19** | T1 | 4.86 | 20.84 | 30.34 | 5.79 | 18.66 | 5.25 | 68.74 | 1.47 |
| **20** | T1 | 8.27 | 9.24 | 9.78 | 0.00 | 0.00 | 0.00 | 0.00 | 43.35 |

| **Subject** | **Time** | **2-heptanone** | **pyridine** | **3-methyl-1-butanol** | **nonanol** | **dimethyl trisulfide** | **1-hexanol** | **6-methyl-5-hepten-2-one** | **2-nonanone** |
| --- | --- | --- | --- | --- | --- | --- | --- | --- | --- |
| **1** | T0 | 18.27 | 72.01 | 0.00 | 0.00 | 1.00 | 0 | 63.98 | 1.21 |
| **2** | T0 | 12.08 | 61.60 | 0.00 | 0.00 | 0.00 | 0 | 12.89 | 1.47 |
| **3** | T0 | 6.20 | 75.74 | 8.99 | 1.53 | 0.00 | 0 | 14.37 | 2.67 |
| **4** | T0 | 16.99 | 83.05 | 4.68 | 1.56 | 1.49 | 0 | 12.62 | 3.70 |
| **5** | T0 | 18.78 | 72.42 | 5.19 | 5.37 | 1.29 | 0 | 3.11 | 2.69 |
| **6** | T0 | 7.50 | 65.60 | 4.93 | 1.05 | 0.00 | 0 | 14.47 | 4.38 |
| **7** | T0 | 6.52 | 67.31 | 0.00 | 0.90 | 2.25 | 0 | 15.65 | 1.46 |
| **8** | T0 | 7.64 | 52.81 | 3.90 | 0 | 0.05 | 0.00 | 11.58 | 3.04 |
| **9** | T0 | 12.80 | 50.60 | 0.88 | 0 | 8.59 | 7.36 | 260.28 | 3.05 |
| **10** | T0 | 24.49 | 88.03 | 1.48 | 0.00 | 1.75 | 0 | 16.40 | 0.00 |
| **11** | T0 | 3.73 | 63.32 | 2.65 | 0 | 5.70 | 11.99 | 14.53 | 3.81 |
| **12** | T0 | 1.04 | 49.87 | 3.19 | 0 | 2.46 | 2.35 | 5.57 | 1.65 |
| **13** | T0 | 7.76 | 75.42 | 0.00 | 4.16 | 1.36 | 0 | 10.72 | 6.10 |
| **14** | T0 | 3.10 | 51.80 | 0.00 | 1.51 | 1.36 | 0 | 4.79 | 1.15 |
| **15** | T0 | 0.00 | 0.00 | 4.39 | 1.01 | 0.00 | 0 | 8.96 | 0.00 |
| **16** | T0 | 10.24 | 50.64 | 0.00 | 0.00 | 1.18 | 0 | 14.98 | 1.92 |
| **17** | T0 | 10.05 | 61.86 | 3.54 | 0.00 | 0.86 | 0 | 36.15 | 3.85 |
| **18** | T0 | 5.59 | 31.72 | 1.42 | 0 | 4.33 | 10.31 | 6.86 | 5.84 |
| **19** | T0 | 1.05 | 58.20 | 1.00 | 0 | 4.39 | 5.65 | 102.44 | 0.00 |
| **20** | T0 | 5.82 | 58.63 | 1.74 | 0.00 | 1.31 | 0 | 149.36 | 1.65 |
| **1** | T1 | 12.02 | 61.20 | 0.00 | 0.99 | 0.00 | 0 | 210.99 | 0.00 |
| **2** | T1 | 18.79 | 65.99 | 0.00 | 0.00 | 0.00 | 0 | 51.01 | 0.00 |
| **3** | T1 | 8.16 | 42.68 | 0.00 | 0.00 | 0.00 | 0 | 121.29 | 2.47 |
| **4** | T1 | 8.94 | 55.10 | 0.00 | 4.35 | 0.00 | 0 | 106.79 | 4.50 |
| **5** | T1 | 19.27 | 71.05 | 1.78 | 2.27 | 1.42 | 0 | 102.48 | 1.35 |
| **6** | T1 | 10.66 | 59.29 | 1.58 | 0.00 | 1.65 | 0 | 204.14 | 1.03 |
| **7** | T1 | 13.76 | 47.68 | 4.33 | 0.00 | 1.36 | 0 | 80.26 | 0.00 |
| **8** | T1 | 7.78 | 17.50 | 4.06 | 0 | 6.78 | 5.79 | 12.26 | 0.00 |
| **9** | T1 | 20.51 | 68.39 | 4.30 | 0 | 8.01 | 4.16 | 62.96 | 3.94 |
| **10** | T1 | 12.87 | 58.14 | 1.14 | 0.00 | 2.10 | 0.00 | 335.78 | 1.42 |
| **11** | T1 | 4.45 | 58.78 | 7.66 | 0 | 4.25 | 42.15 | 14.66 | 1.69 |
| **12** | T1 | 30.21 | 75.70 | 2.20 | 0 | 13.59 | 1.22 | 152.63 | 1.82 |
| **13** | T1 | 7.17 | 34.98 | 2.54 | 1.86 | 1.34 | 0 | 65.13 | 1.65 |
| **14** | T1 | 12.64 | 49.80 | 0.00 | 52.28 | 0.00 | 0 | 81.27 | 0.00 |
| **15** | T1 | 18.78 | 68.36 | 0.00 | 0.00 | 1.57 | 0 | 254.47 | 1.84 |
| **16** | T1 | 17.78 | 67.48 | 3.23 | 0.00 | 1.36 | 0 | 128.64 | 4.90 |
| **17** | T1 | 19.72 | 70.49 | 0.00 | 0.00 | 0.00 | 0 | 7.67 | 3.65 |
| **18** | T1 | 4.44 | 46.00 | 1.38 | 0.00 | 6.90 | 30.79 | 86.45 | 6.28 |
| **19** | T1 | 24.20 | 71.85 | 18.84 | 0.00 | 1.26 | 42.54 | 38.04 | 3.32 |
| **20** | T1 | 22.78 | 80.99 | 1.35 | 0.00 | 0.54 | 0.00 | 134.55 | 2.60 |

| **Subject** | **Time** | **nonanal** | **1-heptanol** | **(E)-2-octenal** | **acetic acid** | **1-octanol** | **propanoic acid** | **isobutyric acid** | **butyric acid** |
| --- | --- | --- | --- | --- | --- | --- | --- | --- | --- |
| **1** | T0 | 26.44 | 5.27 | 0.00 | 9.24 | 0.00 | 119.92 | 15.10 | 41.95 |
| **2** | T0 | 12.49 | 8.91 | 1.12 | 6.53 | 1.65 | 75.30 | 0.00 | 3.08 |
| **3** | T0 | 16.87 | 23.32 | 0.00 | 0.00 | 1.61 | 27.82 | 0.00 | 3.53 |
| **4** | T0 | 32.16 | 52.69 | 0.00 | 6.74 | 2.00 | 131.40 | 0.00 | 12.99 |
| **5** | T0 | 31.63 | 26.06 | 1.65 | 11.48 | 3.18 | 149.86 | 0.00 | 5.27 |
| **6** | T0 | 16.52 | 22.87 | 0.00 | 0.00 | 1.52 | 81.06 | 0.00 | 0.00 |
| **7** | T0 | 19.64 | 3.23 | 0.00 | 3.92 | 0.00 | 37.27 | 0.00 | 10.04 |
| **8** | T0 | 9.85 | 6.20 | 1.04 | 4.22 | 4.85 | 18.51 | 1.08 | 0.54 |
| **9** | T0 | 16.40 | 22.89 | 4.12 | 10.69 | 8.59 | 99.12 | 2.48 | 0.00 |
| **10** | T0 | 30.26 | 0.00 | 2.96 | 12.34 | 1.37 | 87.02 | 0.00 | 149.25 |
| **11** | T0 | 36.67 | 26.43 | 1.15 | 2.29 | 7.75 | 12.29 | 1.55 | 0.84 |
| **12** | T0 | 32.90 | 10.52 | 0.43 | 1.60 | 4.15 | 4.01 | 1.43 | 0.51 |
| **13** | T0 | 24.31 | 49.54 | 0.00 | 3.55 | 0.00 | 93.76 | 0.00 | 15.23 |
| **14** | T0 | 15.62 | 12.55 | 0.00 | 0.00 | 0.00 | 24.31 | 0.00 | 0.00 |
| **15** | T0 | 12.60 | 9.54 | 0.00 | 2.20 | 3.45 | 52.89 | 0.00 | 0.00 |
| **16** | T0 | 25.49 | 9.05 | 2.17 | 7.52 | 0.00 | 83.89 | 0.00 | 0.00 |
| **17** | T0 | 16.22 | 15.89 | 0.00 | 6.19 | 1.35 | 93.34 | 0.00 | 0.00 |
| **18** | T0 | 38.74 | 36.24 | 1.05 | 1.79 | 5.96 | 31.92 | 1.83 | 4.84 |
| **19** | T0 | 12.77 | 57.21 | 2.21 | 1.05 | 4.66 | 21.26 | 2.93 | 10.48 |
| **20** | T0 | 19.78 | 14.52 | 0.98 | 1.34 | 0.00 | 55.82 | 1.90 | 5.06 |
| **1** | T1 | 11.07 | 1.97 | 1.33 | 10.13 | 0.00 | 80.55 | 0.00 | 0.00 |
| **2** | T1 | 24.51 | 8.66 | 0.00 | 8.12 | 0.00 | 102.98 | 0.00 | 68.82 |
| **3** | T1 | 20.81 | 4.47 | 1.63 | 9.19 | 0.00 | 82.76 | 3.46 | 19.98 |
| **4** | T1 | 21.20 | 42.75 | 2.18 | 11.77 | 0.00 | 107.92 | 0.00 | 0.00 |
| **5** | T1 | 26.13 | 12.88 | 1.83 | 9.65 | 2.18 | 81.21 | 0.00 | 7.49 |
| **6** | T1 | 22.70 | 3.70 | 0.00 | 9.44 | 0.00 | 87.68 | 10.44 | 0.00 |
| **7** | T1 | 21.85 | 0.00 | 0.00 | 10.71 | 0.00 | 69.92 | 0.00 | 11.74 |
| **8** | T1 | 121.30 | 44.55 | 1.44 | 2.67 | 8.48 | 25.63 | 4.17 | 1.86 |
| **9** | T1 | 22.19 | 6.59 | 3.12 | 5.63 | 3.89 | 90.46 | 0.01 | 1.01 |
| **10** | T1 | 23.82 | 0.00 | 1.54 | 18.19 | 0.00 | 113.59 | 38.04 | 9.70 |
| **11** | T1 | 29.88 | 14.17 | 0.41 | 1.65 | 7.76 | 13.47 | 1.44 | 1.15 |
| **12** | T1 | 21.40 | 8.37 | 3.92 | 9.40 | 2.54 | 129.00 | 0.00 | 0.00 |
| **13** | T1 | 10.01 | 0.00 | 0.00 | 0.00 | 0.00 | 153.93 | 3.90 | 5.92 |
| **14** | T1 | 19.83 | 5.39 | 1.10 | 6.79 | 0.00 | 89.04 | 0.00 | 0.00 |
| **15** | T1 | 27.49 | 1.46 | 1.87 | 8.44 | 0.00 | 121.71 | 4.29 | 0.00 |
| **16** | T1 | 25.07 | 40.04 | 2.54 | 6.10 | 0.00 | 93.45 | 3.83 | 0.00 |
| **17** | T1 | 18.02 | 13.06 | 2.62 | 9.14 | 1.61 | 155.82 | 1.94 | 0.00 |
| **18** | T1 | 43.22 | 44.04 | 2.79 | 285.54 | 30.42 | 32.13 | 59.74 | 28.13 |
| **19** | T1 | 2.12 | 32.66 | 2.43 | 84.19 | 6.58 | 46.49 | 0.01 | 102.71 |
| **20** | T1 | 25.61 | 8.14 | 4.64 | 7.93 | 0.00 | 86.10 | 3.59 | 5.06 |

| **Subject** | **Time** | **isovaleric acid** | **phenylacetaldehyde** | **thiophene** | **acetophenone** | **valeric acid** | **heptanoic acid** | ***p*-cresol** | **indole** |
| --- | --- | --- | --- | --- | --- | --- | --- | --- | --- |
| **1** | T0 | 6.42 | 0.00 | 25.56 | 0 | 20.60 | 0.00 | 36.38 | 27.63 |
| **2** | T0 | 4.39 | 6.90 | 17.10 | 0 | 1.22 | 0.00 | 1143.18 | 25.69 |
| **3** | T0 | 20.34 | 4.04 | 29.42 | 0 | 26.42 | 0.00 | 870.75 | 20.72 |
| **4** | T0 | 15.05 | 4.16 | 43.10 | 0 | 18.40 | 11.67 | 467.43 | 72.07 |
| **5** | T0 | 22.15 | 14.22 | 40.97 | 0 | 35.23 | 31.59 | 133.99 | 15.74 |
| **6** | T0 | 0.00 | 7.71 | 30.68 | 0 | 16.72 | 32.65 | 914.51 | 72.08 |
| **7** | T0 | 26.49 | 7.38 | 36.66 | 0 | 33.64 | 0.00 | 666.91 | 19.10 |
| **8** | T0 | 24.02 | 33.90 | 48.32 | 13.26 | 24.95 | 0 | 1919.10 | 0.40 |
| **9** | T0 | 38.05 | 4.87 | 34.63 | 6.03 | 37.40 | 0 | 2271.74 | 57.28 |
| **10** | T0 | 12.96 | 20.47 | 36.08 | 0 | 31.74 | 0.00 | 418.36 | 0.00 |
| **11** | T0 | 16.61 | 5.41 | 52.92 | 4.60 | 39.78 | 0 | 1142.05 | 11.19 |
| **12** | T0 | 4.93 | 4.72 | 32.36 | 0.00 | 18.17 | 0 | 2102.13 | 29.31 |
| **13** | T0 | 12.08 | 10.83 | 37.11 | 0 | 30.85 | 39.80 | 254.62 | 19.74 |
| **14** | T0 | 13.88 | 24.13 | 37.12 | 0 | 26.57 | 0.00 | 1343.41 | 34.15 |
| **15** | T0 | 5.81 | 0.00 | 16.57 | 0 | 9.98 | 0.00 | 582.16 | 0.00 |
| **16** | T0 | 12.85 | 9.19 | 30.11 | 0 | 22.89 | 17.94 | 1204.53 | 34.28 |
| **17** | T0 | 28.55 | 6.82 | 21.17 | 0 | 10.74 | 21.17 | 818.07 | 28.60 |
| **18** | T0 | 6.79 | 8.81 | 21.35 | 5.09 | 7.94 | 0 | 987.37 | 15.87 |
| **19** | T0 | 0.00 | 10.54 | 24.77 | 1.33 | 36.65 | 0 | 1873.64 | 39.78 |
| **20** | T0 | 20.37 | 4.09 | 33.28 | 0 | 20.32 | 0.00 | 948.06 | 38.28 |
| **1** | T1 | 0.00 | 44.66 | 16.47 | 0 | 2.73 | 0.00 | 1150.24 | 54.69 |
| **2** | T1 | 7.80 | 5.08 | 24.15 | 0 | 17.80 | 10.65 | 448.18 | 12.96 |
| **3** | T1 | 17.33 | 8.12 | 39.72 | 0 | 20.10 | 6.84 | 1273.09 | 54.10 |
| **4** | T1 | 16.00 | 17.99 | 33.26 | 0 | 9.28 | 49.21 | 1406.13 | 17.31 |
| **5** | T1 | 14.50 | 16.18 | 31.07 | 0 | 30.81 | 9.57 | 301.05 | 30.97 |
| **6** | T1 | 21.02 | 26.71 | 33.16 | 0 | 25.08 | 0.00 | 709.01 | 8.61 |
| **7** | T1 | 16.88 | 11.79 | 35.41 | 0 | 28.55 | 0.00 | 875.94 | 13.09 |
| **8** | T1 | 4.16 | 5.68 | 59.92 | 7.51 | 35.33 | 0 | 1073.29 | 20.57 |
| **9** | T1 | 4.34 | 22.47 | 22.98 | 3.95 | 36.66 | 0 | 1811.26 | 41.63 |
| **10** | T1 | 18.64 | 29.04 | 54.62 | 0 | 42.07 | 8.69 | 1152.23 | 0.00 |
| **11** | T1 | 27.69 | 9.83 | 60.03 | 0 | 26.67 | 0 | 1004.37 | 14.66 |
| **12** | T1 | 66.11 | 27.34 | 47.34 | 0.00 | 25.85 | 0 | 764.61 | 37.04 |
| **13** | T1 | 12.19 | 35.72 | 28.42 | 0 | 18.18 | 0.00 | 963.14 | 0.00 |
| **14** | T1 | 13.17 | 17.82 | 35.89 | 0 | 10.49 | 0.00 | 975.13 | 50.43 |
| **15** | T1 | 15.80 | 43.70 | 37.92 | 0 | 26.55 | 0.00 | 912.98 | 0.00 |
| **16** | T1 | 9.93 | 49.24 | 31.48 | 0 | 22.04 | 63.03 | 694.31 | 32.03 |
| **17** | T1 | 33.33 | 11.75 | 25.59 | 0 | 19.41 | 0.00 | 668.87 | 25.11 |
| **18** | T1 | 29.03 | 41.79 | 5.89 | 3.58 | 16.36 | 0 | 1598.78 | 7.01 |
| **19** | T1 | 34.16 | 8.96 | 0.00 | 4.21 | 4.21 | 0 | 1337.83 | 5.15 |
| **20** | T1 | 9.94 | 7.94 | 21.05 | 0 | 15.34 | 0.00 | 78.56 | 20.30 |
