# Supplementary material for: Impact of a synbiotic food on the gut microbial ecology and metabolic profiles
Source: BMC Microbiol. 2010 Jan 7;10:4. doi: 10.1186/1471-2180-10-4 (PMC2806344; doi:10.1186/1471-2180-10-4)
Supplement: Additional file 2 — Confusion matrix. Confusion matrix derived by 4-fold cross-validation of CAP model obtained using metabolites identified in stool samples collected from 20 volunteers before (T0) and after (T1) the synbiotic food intake. [file 1471-2180-10-4-S2.DOC]

**Additional file 2. Confusion matrix.**

| Observed vs predicted | T0 | T1 |
| --- | --- | --- |
| T0 | 20/20 | 0/20 |
| T1 | 0/20 | 20/20 |
| Prediction rate % | 100 | 100 |
